# The prognostic value of stem cell markers in triple-negative breast cancer

**DOI:** 10.3389/pore.2023.1611365

**Published:** 2023-12-22

**Authors:** Szintia Almási, Ágnes Nagy, Tibor Krenács, Tamás Lantos, Tamás Zombori, Gábor Cserni

**Affiliations:** ^1^ Department of Pathology, Albert Szent-Györgyi Medical Centre, University of Szeged, Szeged, Hungary; ^2^ Department of Pathology and Experimental Cancer Research, Faculty of Medicine, Semmelweis University, Budapest, Hungary; ^3^ Department of Medical Physics and Informatics, University of Szeged, Szeged, Hungary; ^4^ Department of Pathology, Bács-Kiskun County Teaching Hospital, Kecskemét, Hungary

**Keywords:** immunohistochemistry, prognosis, CD44, ALDH1, stem cell markers, triple-negative breast cancer

## Abstract

Among the many consecutive theories of cancer, the stem cell theory is currently the most accepted one. Cancer stem cells are located in small niches with specific environment, renew themselves and are believed to be responsible for many recurrences. They can be highlighted with stem cell markers, but often these markers also label tumor cells, and this may represent a phenotypical change associated with prognosis. In this study, we attempted to match tumor outcomes with the expression of the following stem cell markers: ALDH1, AnnexinA1, CD44, CD117, CD166, Nanog and oct-4. Tissue microarray blocks from triple-negative breast cancers were immunostained for the listed markers, and their expression by the majority of tumor cells (diffuse positivity) was correlated with prognosis. Of the 106 tumors investigated, diffuse positivity was seen in 7 (ALDH1), 33 (AnnexinA1), 53 (CD44), 44 (CD117 membranous only), 49 (CD117), 72 (CD166), 19 (Nanog), and 11 (oct-4) cases. With a median follow-up of 83 months, ALDH1 and CD117 expression was associated with DFS, whereas CD44, CD117 and CD166 were associated with OS estimates, based on Kaplan-Meier analyses. In the multivariate Cox proportional hazard models (including the examined markers and clinicopathological data which had a statistical impact in the univariate analysis), the pN category and the lack of ALDH1 expression were independent prognosticators for DFS, and the pN category and diffuse CD44 staining were independent prognosticators for OS. In the multivariate analysis including all of the examined clinicopathological data and markers, only CD117 showed a statistical impact on OS. We failed to demonstrate a prognostic impact for most stem cell markers tested in triple-negative breast cancer, but lack of ALDH1 staining and CD44 expression appears as of prognostic value, requiring further examination in independent studies.

## Introduction

Among the many consecutive theories of cancer, the stem cell theory is currently the most accepted one. Carcinomas harbor a set of stem cells from which the tumor originates; these are located in small niches with a specific environment, renew themselves and are believed to be responsible for many recurrences [1], whereas the major part of the tumor bulk is composed of more differentiated tumor cells [1]. Tumor stem cells can be highlighted by stem cell markers, but the antibodies used to highlight these markers are not specific for stem cells and often highlight other cell populations along with them [1]. Immunostaining with stem cell marker antibodies may represent a phenotypical change associated with prognosis [1]. In this study, we attempted to match tumor outcomes with stem cell marker expression by the majority of tumor cells. The following markers were assessed.

Aldehyde dehydrogenase-1 (ALDH1) is a member of the aldehyde dehydrogenase enzyme superfamily [2]. ALDH enzymes are localized in the cytosol, nucleus, endoplasmic reticulum and also in the mitochondria [2]. These enzymes are involved in the metabolism of aldehyde, which can be exogenous or can be produced inside the body, for example, by lipid peroxidation or amino acid catabolism. ALDHs can protect the cells from the toxicity of active aldehydes by catalyzing their oxidation [2]. ALDH1 can be used to identify not just normal stem cells, but also cancer stem cells, and it is also a dependable marker of cancer stem cells in a variety of solid tumors, including cancers of the breast, head and neck, liver, pancreas, colon, lung, cervix, ovary, prostate and bladder [2, 3]. Additionally, ALDH1 can be used as a marker for normal and malignant stem cells both in the mammary gland and the colon [2]. ALDH1 is expressed in normal colonic and gastric epithelium, cells of the liver and pancreas cells and can also be expressed in lung tissue [2]. ALDH1A1 and ALDH32, both play an important role in cell self-protection, cellular expansion and differentiation. In breast cancer, ALDH1 staining would highlight both normal and malignant stem cells [2, 3]. This marker can be used efficiently to identify cancer stem cells in tissues which do not express ALDH1 at high levels under normal circumstances, for example, in the breast [2, 3].

AnnexinA1 is a calcium-dependent phospholipid-binding protein, and also known as lipocortin 1, the first member of the Annexin superfamily [4]. AnnexinA1 is located in the cytoplasm, or it can bind to cytoskeletal proteins and then it can control the communication between the cell and the extracellular matrix, alternately it can be located in the nucleus, and when the cell is finally activated, it will be moved to the cell surface [4]. AnnexinA1 has an important role in pro-inflammatory and anti-inflammatory response, cell proliferation, differentiation, metastasis formation and also in the apoptotic process [4]. This marker is expressed in gastrointestinal tissues, and a wide range of malignancies, like esophageal, gastric, colorectal, biliary, pancreatic cancers, lung adenocarcinomas and also gliomas [4]. AnnexinA1 expression in cancers is not just tissue-specific but can be different in different types of cancers of the same tissue, for example, it is differently expressed in primary invasive breast cancer, *in situ* breast carcinoma and metastatic breast cancer. In the first two, the expression is generally reduced, but in the latter, it is significantly increased. In benign breast epithelium, AnnexinA1 expression can be variable [4]. AnnexinA1 can promote the invasiveness of tumor cells by activating the Transforming Growth Factor-β (TGFβ) and Nuclear factor kappa B (NF-κB) pathways. The activation of the TGFβ pathway will lead to epithelial-to-mesenchymal transition in cancer cells. Both AnnexinA1 and A2 are mediators of the endocytosis of epidermal growth factor receptor (EGFR), and it is known that EGFR activity is related to breast cancer progression. Of note, EGFR expression is also a marker of the basal-like phenotype in triple-negative breast cancers [4–6].

Cluster of differentiation 44 (CD44) is considered a cell surface glycoprotein receptor, which mainly binds hyaluronic acid, and this linkage takes part in cell invasiveness [7]. CD44 can also be connected with a cell adhesion molecule, an integrin which takes part in signaling between cells, cell proliferation and cell differentiation [7]. It has a short standard form (CD44s) and several isoforms as a result of alternative splicing (CD44v) [8]. CD44 has an important role in cell-cell and cell-matrix communication [7]. This receptor is expressed in a wide range of cell types, for example, in leukocytes, epithelial cells, mesodermal cells and also in numerous cancer stem cells [7]. Cancer stem cells produce hyaluronic acid to invoke tumor-associated macrophages [7]. Both cells can synthesize platelet-derived growth factor (PDGF), which keeps the tumor cells in a constant proliferative phase, this collaboration leads to stromal cells gathering into the cancer stem cell niche, and the stromal cells produce growth factors which coordinate stem cell activity and reproduction [7]. CD44 expression on breast cancer cell surface is increased compared to normal breast epithelial cells [9].

Cluster of differentiation 117 (CD117) also known as c-Kit, is a proto-oncogene or stem cell factor receptor; it is a transmembrane receptor tyrosine kinase, and as a ligand, stem cell factor (SCF) can bind to it [10]. CD117 is expressed in progenitor cells, for example, in the breast, in the hemopoietic system, in the myocardium, in the lung, in the testis and it is also expressed in luminal epithelial cells in adult normal breast tissue [10]. CD117 can become oncogenic either by being overactivated or by constitutive mutations leading to CD117 being activated without a ligand, and as a consequence, cell proliferation, differentiation and migration will be upregulated [11]. The inactive c-Kit is located on the cell surface, and when SCF binds to the receptor, autophosphorylation occurs [11]. This process will promote signal transduction pathways, for example, in the Janus kinase/signal transducer and activator of transcription (JAK/STAT), rat sarcoma virus/mitogen-activated protein (RAS/MAP) kinase pathways [11]. By triggering these pathways, CD117 has an important role in cell proliferation, differentiation, apoptosis and motility [11]. CD117 can be used as a marker to detect hemopoietic stem cells and cancer stem cells by immunohistochemistry, and the staining is positive when seen on the cell membrane, but also if present in the cytoplasm [10–12]. CD117 expression has also been linked to the basal-like phenotype in a study searching for immunohistochemistry (IHC) based surrogate markers of this intrinsic subtype, but cytokeratin 5 (CK5) and EGFR were found superior [5].

Cluster of differentiation 166 (CD166), also known as activated leukocyte cell adhesion molecule (ALCAM), was identified as a cell surface glycoprotein on the surface of activated leukocytes [13]. It is a prognostic marker for various tumors and correlates with poor outcome [13]. CD166 can be found in epithelial cells, myeloid progenitor cells, vascular endothelial cells, and also a variety of stem cells, including cancer stem cells [13]. CD166 can be expressed in invasive cancers, such as breast, lung, bladder, prostate, liver, pancreas, head and neck cancers, as well as epithelial ovarian carcinomas, [13]. CD166 has a role in interaction between cells, like epithelial or endothelial cells and lymphocytes, and in angiogenesis [13]. CD6, CD9, S100B and galectin-8 are ligands for CD166 in breast tumors and endothelial tumor cells. The localization of CD166 is different in normal breast epithelial cells and breast tumor cells [13]. In normal cells, CD166 expression is increased on the cell surface, and the expression is decreased in the cytoplasm [13].

Nanog is a stem cell transcription factor, which has a role in cell differentiation, the apoptotic process and cell fate determination [14]. It is involved in the preservation of pluripotency in embryonic stem cells [14]. Under physiological circumstances, during life, Nanog expression is at a low level in a wide range of tissues, in contrast to cancer stem cells, where Nanog expression can be increased [15]. Nanog can be expressed in the ovaries, the testis, the small intestines, the thyroid gland and also in the glandular cells of the uterine cervix [15]. Despite this fact, Nanog is not expressed in most of the normal tissues [15]. The expression of this marker can be seen as nuclear and cytoplasmic positivity in different types of precancerous lesions and invasive cancers, for example, in head and neck squamous cell dysplasias and carcinomas, salivary gland mucoepidermoid carcinomas, gliomas, lung, colorectal, gastric, esophageal, renal cell and urothelial carcinomas, furthermore in testicular germ cell tumors and ovarian cancers [15]. In breast cancer, it shows rather nuclear than cytoplasmic positivity [15]. Nanog expression positively correlates with proliferation, and also with higher stage, worse patient outcome, poor differentiation, and according to some studies, the expression correlates with therapy resistance [16].

Octamer binding transcription factor 4 (oct-4) is a homeodomain transcription factor of the Pit-Oct-Unc (POU) family, and it has an important role in the regulation of pluripotency in somatic cells, embryonic stem cells and in cancer stem cells [17]. A wide range of adult stem cells, such as breast epithelial, kidney-, mesenchymal, liver- and pancreatic stem cells express oct-4 [18]. Oct-4 expression can be found in bladder, ovarian, prostate, rectal cancers, gliomas, medulloblastoma, melanoma, hepatocellular carcinoma, esophageal squamous cell carcinoma and acute myeloid leukemia [18]. When breast epithelial stem cells start their differentiation, oct-4 expression decreases [18]. In normal breast tissue, and also in breast carcinoma, IHC demonstrates nuclear and cytoplasmic staining [18].

## Materials and methods

We examined triple-negative breast cancers (TNBCs) surgically treated at the Bács-Kiskun County Teaching Hospital between 2005 and 2017. By definition, all the tumors lacked estrogen (ER) and progesterone receptor (PR) and human epidermal growth factor receptor 2 (HER2) expression. From the selected formalin-fixed, paraffin-embedded tumor samples, tissue microarray (TMA) cores were taken. In each case, two, three or four cores were taken from the donor blocks. One TMA contained 7 rows, and 10 columns, therefore, on a single TMA slide, we could examine 70 TNBC core samples.

Four-µm-thick TMA serial sections of the TNBC series were immunostained using either the Roche-Ventana BenchMark (Tucson, AZ, USA) automated instrument with high pH antigen retrieval (in CC1 buffer) for 60 min and the Ultraview detection system for 20 min; or using a manual procedure at room temperature, including routine dewaxing and rehydration, antigen retrieval in pH 9.0 Tris (0.1 M)-EDTA (0.01 M) buffer using an electric pressure cooker (Avair, Biofa, Veszprém, Hungary) for 20 min. and incubations using the primary antibodies overnight followed by a micropolymer detection system (Histols, Histopathology Ltd. Pecs, Hungary) for 60 min. Finally, both types of reactions were developed using DAB-hydrogen peroxide, resulting in a brown staining. The following human antigen-specific antibodies were used: mouse monoclonal anti-ALDH1 (1:75; clone 44, CellMarque, Merck, Darmstadt, Germany); anti-Annexin1 (1:200, clone MRQ-3), anti-CD44 (1:50; clone MRQ13), anti-oct-4 (1:20-35; clone MRQ-10; all three CellMarque, Rocklin, CA, USA); and anti-CD166 (1:100, clone MOG/07, Novocastra, Newcastle upon Tyne, UK); rabbit monoclonal anti-Nanog (1:35, clone EP225, CellMarque); as well as rabbit polyclonal anti-CD117 (manual 1:100 or Ventana 1:700, RB-9038, Thermo Fisher, LabVision, Fremont, CA, USA). Immunostained TMA slides were digitalized using a Pannoramic 1000 whole slide scanner (3DHistech Ltd. Budapest, Hungary) and analyzed using a visual scoring system.

**FIGURE 1:**
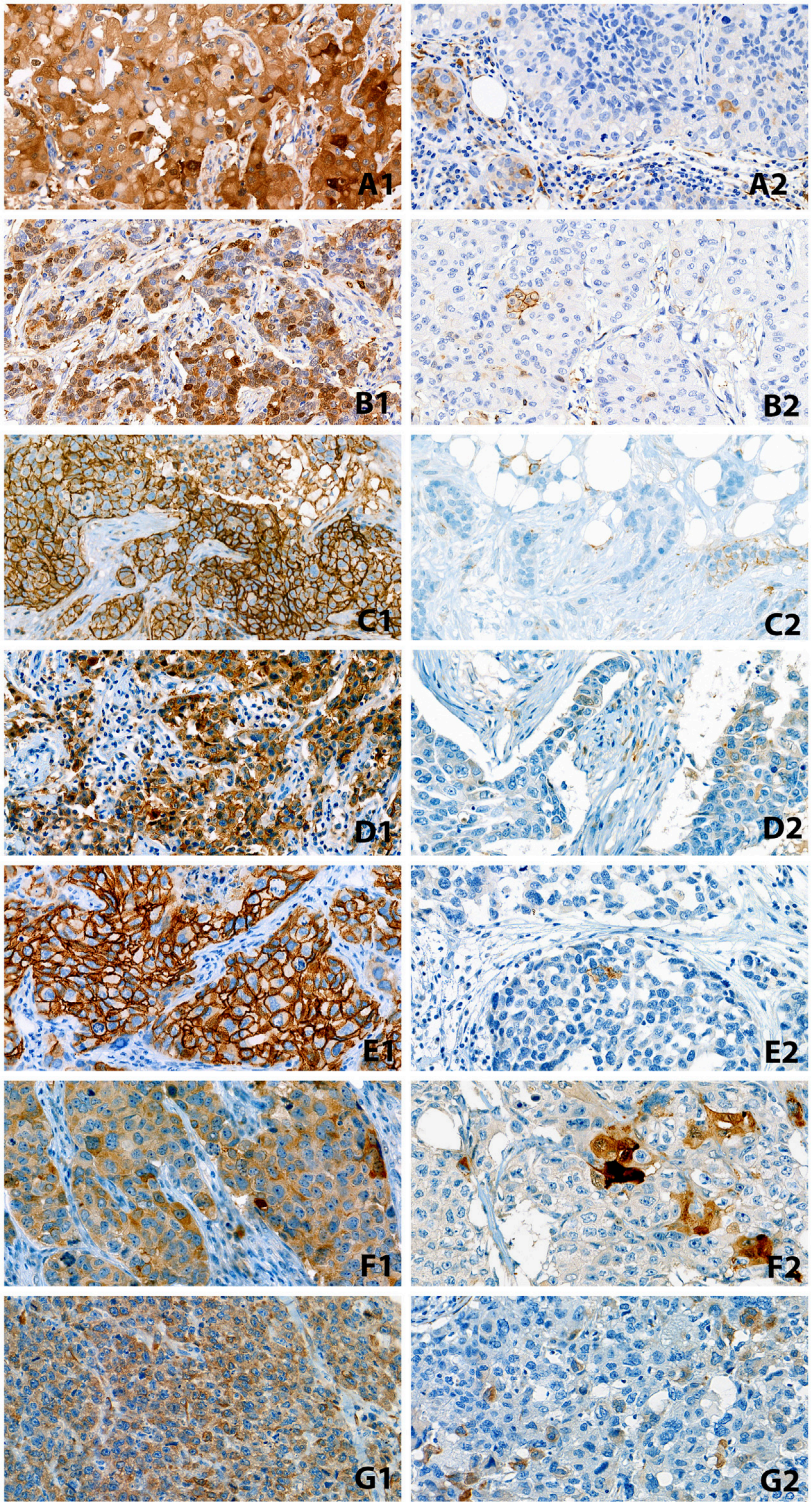
Examples of diffusely (1) and focally (2) positive staining with the studied markers. **(A)**: ALDH1, **(B)**: AnnexinA1, **(C)**: CD44, **(D)**: CD117, **(E)**: CD166, **(F)**: Nanog, **(G)**: oct-4 (All ×40 magnification).

The scanned slides were evaluated by two observers, SzA and GC. For each marker, the proper localization of the staining (e.g., nuclear, cytoplasmic or membranous) of any intensity in at least 50% of the tumor cells was interpreted as diffusely positive, labelling between 1% and 50% was considered focally positive, and cases with no staining at all or false staining with non-proper localization were interpreted as negative. Accordingly, we distinguished between 3 rough patterns of staining: negative, focal and diffuse. For AnnexinA1, CD44, CD117 and CD166, cytoplasmic and membranous expression, while for ALDH1, Nanog and oct-4 IHC, nuclear and/or cytoplasmic stainings were considered positive. CD117 was also evaluated as a membranous staining only (CD117*). Examples of positive stainings are depicted in Figure 1.

The Spearman rank correlation test was used for the analysis of marker expression and different clinicopathological variables. After the evaluation, we compared the different marker expressions for overall survival (OS) and disease-free survival (DFS). Treatment and follow-up-related data were taken from the patients’ digital charts. For the statistical analyses, and generation of the Kaplan-Meier curves, the SPSS (IBM, SPSS 23.0, Armonk, NY, USA) package was used. The log-rank test was utilized when comparing survival curves. The level of significance was set at *p* < 0.05. Patients lost to follow-up or alive at the last follow-up were censored at the time of the last visit. Stem cell markers with a statistical impact on survival were entered into a multivariate model including known prognosticators of breast cancer, such as tumor size, nodal status and histological grade, and the Cox proportional hazard model was applied to check whether any had an independent effect on the outcome. For the evaluation of the differences between the marker expression and OS, DFS we performed the chi-square (Fisher’s exact) test with Bonferroni-Holm correction. This correction compensates for repetitive multiple pairwise tests and leads to the modification of the original *p* < 0.05 threshold for significance. As most events with high-grade TNBCs occur within 5 years, 5 year survival estimates have been chosen to reflect the outcome of subgroups according to stem cell marker expression.

## Results

### Study population

Altogether TMAs from 106 female TNBCs were selected. The clinicopathological characteristics of the examined cases are presented in Table 1. The median age of the patients was 59 years. The majority of the cases included were invasive breast carcinomas of no special type, with the addition of 5 metaplastic and 3 mixed micropapillary carcinomas. None of the tumors was well differentiated. Most tumors were of the pT1 and pN0 categories and were treated by breast conservation and sentinel lymph node biopsy. The majority of the patients received adjuvant chemotherapy and radiotherapy as summarized in Table 1. Most of the patients got chemotherapy in the adjuvant setting, while 7 patients had neoadjuvant chemotherapy (these latter were included in the series as no substantial regression occurred in their disease). Altogether, 14 patients did not receive any treatment besides surgery, either based on their known comorbidities and relative contraindications or the rare refusal to all forms of further therapy.

**TABLE 1:** Clinicopathological parameters of the studied cases.

		Value (range)
Median age (years)		59 (29–81)
Median tumor size (mm)		20 (7–107)
Histological type		
	NST	98
	Mixed micropapillary	3
	Metaplastic	5
pT category		
	pT1	55
	pT2	43
	pT3	4
	pT4	4
pN category		
	pN0	57
	pN1	37
	pN2	10
	pN3	2
Grade		
	1	0
	2	5
	3	101
Surgery type		
	Breast-conservative	80
	Mastectomy	26
	SNB	56
	SNB and ALND	24
	ALND	25
	No axillary surgery	1
Chemo and Radiotherapy		
	Taxane containing therapy	67
	Anthracyclin containing therapy	12
	CMF	4
	RT	83
	Only RT without Chemotherapy	9
	Neither chemotherapy nor RT	14

NST: no special type breast cancer, SNB: sentinel lymph node biopsy, ALND: Level I-II axillary lymph node dissection, CMF: cyclophosphamide, methotrexate, and fluorouracil, RT: radiotherapy

The median follow-up of the cases was 83 months (range: 5–209), with median DFS and OS of 62 (range: 1–203) and 83 (range: 5–209) months, respectively. There were 36 deaths (21 due to breast cancer) during the follow-up period, in addition, 20 patients had recurrent disease, whereas 50 were with no evidence of disease at the last follow-up.

### Stem cell marker expression

The stem cell marker expression showed no correlation with clinicopathological variables (patient age, tumor size and grade, pT or pN categories) except for three (Supplementary Table S1). A positive correlation was found between ALDH1 expression and pT categories (*p* = 0.024). CD166 expression showed a positive correlation with tumor grade (*p* = 0.045), and patient age (*p* = 0.044). The Fisher’s exact test showed an association between CD166 expression and tumor grade (*p* = 0.038) (Supplementary Table S2). Of the 98 cases with evaluable CD166 staining, 95 were of Grade 3 and 71, 16, and 8 showed diffuse, focal, and no CD166 staining, respectively. Two grade 2 TNBCs showed no staining, and one was diffusely positive for this marker. The proportion of cases staining with each stem cell marker and the associated 5 year survival estimates are reported in Supplementary Table S3. Based on the results there were no significant differences in survivals according to stem cell marker expression; focal or diffuse cytoplasmic and membranous CD117 expressing cases showed marginally significant differences in DFS: 22/49 with a diffuse staining pattern and 22/31 with a focal staining pattern were with no evidence of disease after 5 years (Supplementary Table S3).

**FIGURE 2:**
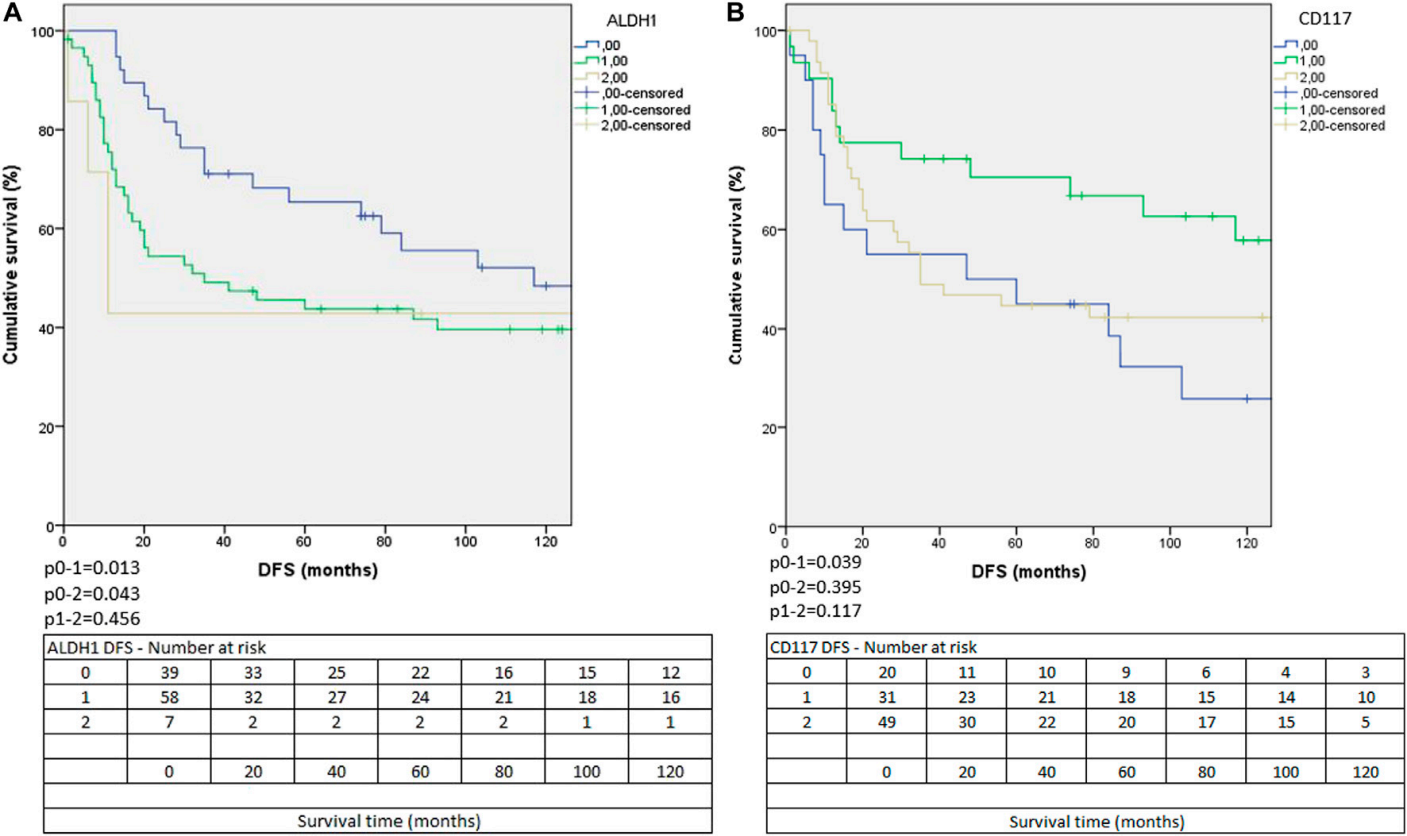
The Kaplan-Meier curves of ALDH1 **(A)** and CD117 membranous and/or cytoplasmic expressions **(B)** according to DFS. (0: negative, 1: focally positive, 2 diffusely positive staining).

**FIGURE 3:**
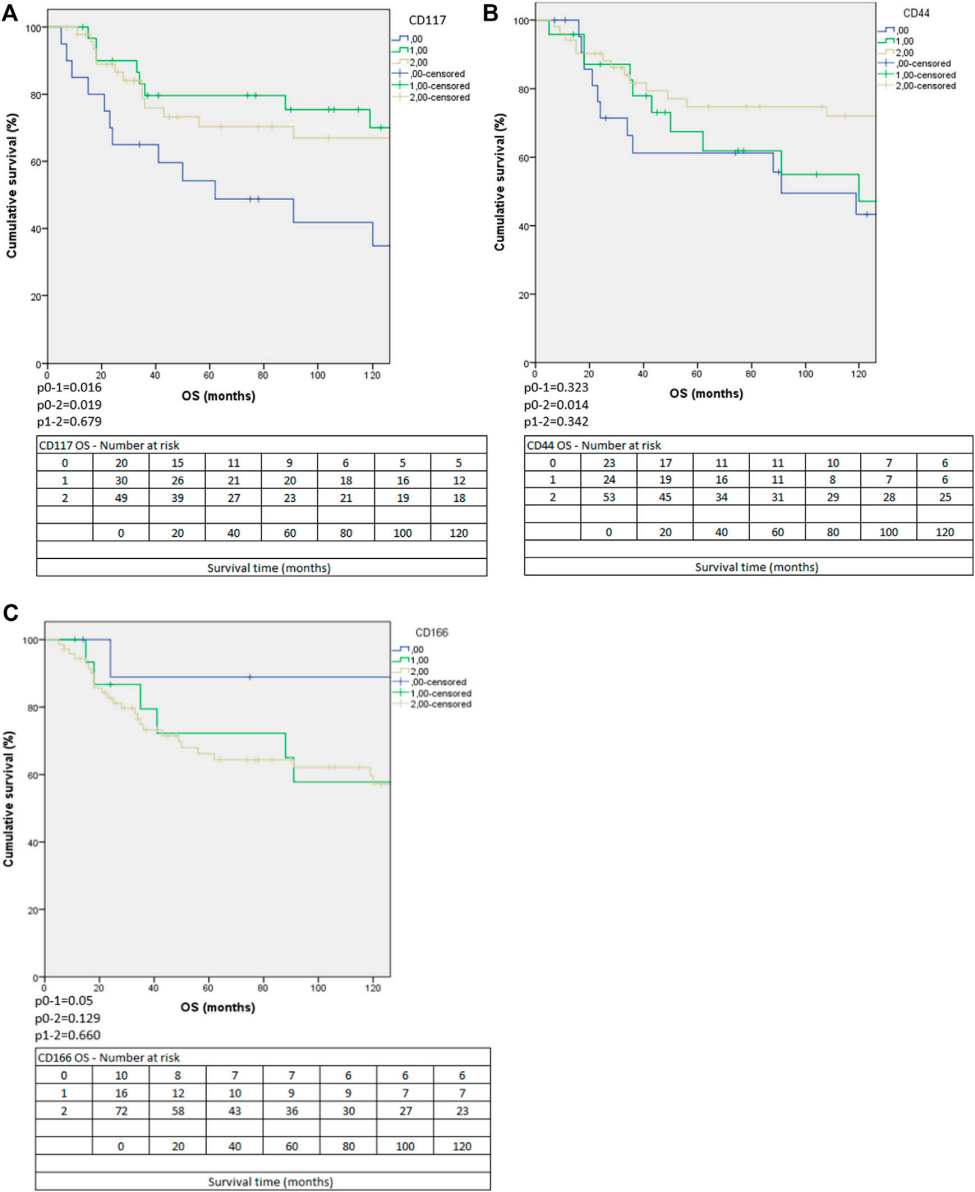
The Kaplan-Meier curves of membranous and/or cytoplasmic expression of CD117 **(A)**, CD44 **(B)** and CD166 **(C)** expressions according to OS. (0: negative, 1: focally positive, 2 diffusely positive staining).

For DFS, only ALDH1 and CD117 (with either membranous or cytoplasmic staining) had different survivals according to their staining patterns based on the Kaplan-Meier analysis (Figures 2A, B), whereas for OS, we noted differences in survival according to the stainings of CD44, CD117 (with either membranous or cytoplasmic staining) and CD166 (Figures 3A–C).

In ALDH1-negative tumors, a significantly better DFS was seen compared to the cases with focal, (*p* = 0.013) or diffuse positivity (*p* = 0.043). In the focally or diffusely positive cases, the DFS was similar (*p* = 0.456) (Figure 2A). The OS was the highest in ALDH1 negative cases, and the diffuse positive cases had the shortest OS, but the differences were not significant (*p* = 0.208). ALDH1 positivity showed a negative prognostic effect.

For CD117, only combined membranous and/or cytoplasmic staining categories showed differences in survival according to the Kaplan-Meier analysis. For DFS, cases with focal staining had better survival than negative cases (*p* = 0.039), but cases with diffuse staining did not differ significantly from the other two groups (negative cases compared to diffusely positive cases: *p* = 0.395, focally positive cases compared to diffusely positive cases *p* = 0.117) (Figure 2B). For OS, cases with no staining had worse survival than cases with either focal (*p* = 0.016) or diffuse (*p* = 0.019) staining. Cases with focal or diffuse staining had similar OS (Figure 3A). According to these results, CD117 might have prognostic value.

Concerning CD44, a significant difference was seen between the OS of negative and diffusely positive cases (*p* = 0.014) with better survival for the latter group. Interestingly the OS of focally positive cases was nearly the same as that of the negative cases (Figure 3B). We found no significant difference according to the OS between the focally and diffusely positive cases (*p* = 0.342).

Regarding CD166 staining, a marginally significantly worse OS was identified for cases with focal staining, when compared to the negative cases (*p* = 0.05). Interestingly, the cases with diffuse marker expression showed no statistical significance from negative cases, which can probably be explained by the low case number in the negative group (Figure 3C).

According to the univariate Cox regression analysis for OS (Table 2) the pT category (HR: 6.398), the pN category (HR: 3.607) and the diffuse CD44 expression (HR: 0.428) had a statistical impact. For DFS (Table 3) the pT category (pT3: HR: 6.008; pT4: HR: 5.497), the pN category (pN2: HR: 2.708; pN3: HR: 19.754) and the focal ALDH1 expression (HR: 1.889) had statistical impact. Based on the multivariate Cox regression analysis including those clinicopathological data and marker expressions which had a statistical impact in the univariate analysis (Table 4), for OS, only CD44 staining (HR: 0.386) and the pN category (HR: 10.179) had an independent impact on prognosis. For DFS, only the pN category (HR: 12.041) and among the stem cell markers ALDH1 (HR: 1.832) were found to be of independent prognostic impact. In addition, in the multivariate Cox regression analysis for both DFS and OS including all the examined markers and clinicopathological data, we found that pT and pN categories had a statistical impact on DFS (pT3: HR: 16.835; pN3: HR: 11.365) (Table 3). Interestingly, based on this analysis only CD117 diffuse positivity showed statistical impact on OS (HR: 0.076) (Table 2).

**TABLE 2:** The results of the univariate and multivariate Cox regression analysis for OS.

	Univariate analysis			Multivariate analysis		
	HR	95% CI	*p*	HR	95% CI	*p*
pT						
1	reference			reference		
2	1.103	0.581–2.092	0.763	0.836	0.348–2.008	0.689
3	4.027	0.890–18.219	0.070	1.228	0.062–24.257	0.892
4	**6.398**	**1.406–29.106**	**0.016**	0.795	0.092–6.836	0.835
pN						
0	reference			reference		
1	0.918	0.445–1.894	0.817	1.336	0.463–3.854	0.591
2	**3.607**	**1.512–8.603**	**0.004**	1.592	0.348–7.268	0.548
3	7.211	0.919–12.583	0.060	9.289	0.840–102.707	0.069
Grade						
2	reference			reference		
3	1.308	0.315–5.429	0.700	0.317	0.010–10.001	0.514
ALDH1						
Negative	reference			reference		
Focal	1.591	0.813–3.114	0.175	2.602	0.773–8.754	0.122
Diffuse	2.187	0.621–7.708	0.223	1.845	0.254–13.381	0.544
AnnexinA1						
Negative	reference			reference		
Focal	1.943	0.584–6.463	0.278	1.914	0.433–8.459	0.391
Diffuse	1.693	0.481–5.952	0.411	2.080	0.452–9.566	0.346
CD44						
Negative	reference			reference		
Focal	0.636	0.277–1.459	0.285	0.669	0.210–2.134	0.497
Diffuse	**0.428**	**0.206–0.888**	**0.022**	0.460	0.154–1.373	0.164
CD117*						
Negative	reference			reference		
Focal	0.577	0.246–1.350	0.205	0.225	0.028–1.776	0.157
Diffuse	0.764	0.377–1.551	0.457	2.025	0.186–21.978	0.561
CD117**						
Negative	reference			reference		
Focal	0.377	0.165–1.345	0.078	0.615	0.082–4.589	0.635
Diffuse	0.441	0.212–1.331	0.104	**0.076**	**0.006–0.929**	**0.043**
CD166						
Negative	reference			reference		
Focal	3.699	0.794–17.226	0.095	9.020	0.313–259.600	0.199
Diffuse	3.131	0.740–13.244	0.120	11.974	0.472–303.720	0.132
Nanog						
Negative	reference			reference		
Focal	0.907	0.384–2.140	0.823	0.404	0.112–1.447	0.164
Diffuse	1.414	0.647–3.092	0.384	1.491	0.542–4.099	0.438
oct-4						
Negative	reference			reference		
Focal	0.866	0.425–1.764	0.692	0.873	0.309–2.464	0.798
Diffuse	1.672	0.652–4.287	0.283	2.904	0.680–12.388	0.149

Membranous (*); membranous and/or cytoplasmic (**) immunostaining.

Significant variables are highlighted in bold.

**TABLE 3:** The results of the univariate and multivariate Cox regression analysis for DFS.

	Univariate analysis			Multivariate analysis		
	HR	95%CI	*p*	HR	95%CI	*p*
pT						
1	reference			reference		
2	1.029	0.595–1.781	0.915	0.839	0.411–1.711	0.629
3	**6.008**	**1.749–20.635**	**0.004**	**16.835**	**1.539–184.083**	**0.020**
4	**5.497**	**1.881–16.058**	**0.001**	2.200	0.402–12.027	0.362
pN						
0	reference			reference		
1	1.249	0.702–2.220	0.448	1.007	0.448–2.262	0.986
2	**2.708**	**1.224–5.991**	**0.013**	1.045	0.265–4.111	0.949
3	**19.754**	**4.031–96.790**	**<0.001**	**11.365**	**1.290–100.067**	**0.028**
Grade						
2	reference			reference		
3	1.037	0.324–3.318	0.950	4.273	0.359–50.843	0.250
ALDH1						
Negative	reference			reference		
Focal	**1.889**	**1.052–3.077**	**0.044**	2.539	1.032–6.245	0.042
Diffuse	1.775	0.602–5.230	0.297	2.858	0.633–12.894	0.171
AnnexinA1						
Negative	reference			reference		
Focal	0.931	0.425–2.037	0.858	0.665	0.245–1.804	0.424
Diffuse	1.063	0.467–2.420	0.884	0.828	0.282–2.427	0.731
CD44						
Negative	reference			reference		
Focal	0.637	0.306–1.326	0.227	0.626	0.233–1.676	0.351
Diffuse	0.625	0.337–1.162	0.137	0.582	0.237–1.431	0.238
CD117*						
Negative	reference			reference		
Focal	0.668	0.315–1.415	0.292	0.859	0.136–5.408	0.872
Diffuse	1.136	0.624–2.066	0.675	0.730	0.129–4.118	0.722
CD117**						
Negative	reference			reference		
Focal	0.453	0.212–1.101	0.061	0.472	0.081–2.737	0.403
Diffuse	0.758	0.400–1.438	0.397	0.524	0.080–3.425	0.500
CD166						
negative	reference			reference		
Focal	1.670	0.594–4.696	0.330	1.390	0.329–5.863	0.653
Diffuse	1.221	0.479–3.111	0.674	0.743	0.201–2.746	0.656
Nanog						
Negative	reference			reference		
Focal	0.959	0.481–1.910	0.906	0.701	0.268–1.829	0.468
Diffuse	1.092	0.548–2.174	0.801	1.650	0.714–3.811	0.240
oct-4						
Negative	reference			reference		
Focal	0.624	0.347–1.123	0.115	0.802	0.358–1.798	0.592
Diffuse	0.911	0.397–2.091	0.827	0.684	0.208–2.254	0.533

Membranous (*); membranous and/or cytoplasmic (**) immunostaining.

Significant variables are highlighted in bold.

**TABLE 4:** The results of the multivariate Cox regression analysis for OS and DFS, including the markers which showed statistical impact on survival in the univariate Cox regression analysis.

	OS			DFS		
	HR	95%CI	*p*	HR	95%CI	*p*
pT						
1	reference			reference		
2	0.894	0.460–1.737	0.741	0.877	0.500–1.538	0.647
3	1.369	0.150–12.426	0.779	2.768	0.454–16.868	0.269
4	1.155	0.190–7.006	0.875	2.167	0.505–9.298	0.297
pN						
0	reference			reference		
1	0.953	0.4462.036	0.901	1.140	0.634–2.053	0.659
2	3.367	1.210–9.371	0.020	1.895	0.614–5.848	0.266
3	**10.179**	**1.143–90.640**	**0.037**	**12.041**	**2.036–71.210**	**0.006**
ALDH1						
Negative				reference		
Focal				**1.832**	**1.014–3.410**	**0.048**
Diffuse				2.029	0.655–6.288	0.219
CD44						
Negative	reference					
Focal	0.665	0.279–1.582	0.356			
Diffuse	**0.386**	**0.172–0.862**	**0.020**			

Significant variables are highlighted in bold.

## Discussion

Of the 7 examined stem cell markers in TNBCs, the Kaplan-Meier analyses suggested a prognostic value only for ALDH1, CD117, CD44 and CD166, whereas the multivariate analysis reduced this to ALDH1 (for DFS) and CD44 (for OS). Although not numerous, several previous studies have examined the prognostic impact of stem cell marker expression in breast cancers.

We experienced no prognostic associations with AnnexinA1, Nanog and oct-4, but there are some publications with opposite results. Wang et al. analyzed the prognostic value of AnnexinA1 expression in TMAs of 135 invasive breast carcinomas; lack of AnnexinA1 expression (0 to <5% staining) correlated with pathological TNM stage and especially with lymph node metastases [19]. AnnexinA1 expression correlated with better OS estimates, but such an association was not seen with DFS estimates [19].

Nagata et al. examined the correlation between pluripotent stem cell-inducing factor expression and the prognosis of breast cancer in 100 cases [20]. Among the cases examined, there were ER-positive and HER2-negative, ER-negative and HER2-positive and also triple-negative (TN) cases [20]. They found a significant association between high Nanog expression and worse OS and DFS (*p* = 0.033, *p* = 0.004), but 8 of the 9 high Nanog-expressing tumors were ER-positive [20]. No significant differences in DFS or OS were found according to oct-4 expression [20]. Another study also found that in ER+ tumors, oct-4 expression was related to a worse outcome and resistance to tamoxifen [17], but the lack of ER expression in the present series does not make the results comparable.

According to Wang et al., Nanog and oct-4 are associated with poor prognosis among breast cancer patients [21]. They examined 126 breast cancers inclusive of 19 with basal-like phenotype. 52 cases (15 basal-like tumors) were positive for oct-4 and 47 (14 basal-like carcinomas) for Nanog with 26 cases (12 basal-like cancers) co-expressing the two markers [21]. (Their definition of IHC positivity, ≥1%, corresponds to a category inclusive of our focally and diffusely staining categories.) With multivariate analysis, there was an association between oct-4 or Nanog expression and the presence of lymph node metastasis (*p* = 0.003], and the molecular type of breast cancer (*p* = 0.001) [21]. Cumulative survival was shorter in all cases expressing either of the markers than in patients with dual negative tumors [21].

In our study, the CD117 negative cases had significantly shorter OS than the positive ones, but the multivariate analysis did not find this to be of independent effect. A similar effect was noted for DFS, too, but again, this did not turn out to be of significance in the multivariable analysis. Luo et al examined the cytoplasmic or membranous CD117 expression in 58 TNBC and 48 non-TNBC, of which the first set showed a significantly greater percentage of staining (48% vs. 29%) [22]. CD117 positivity was associated with tumor recurrence and some markers of poor prognosis (vascular invasion, proliferation) [22]. Tsutsui et al. also examined the association between CD117 expression and prognosis [23]. They investigated 217 no special type invasive breast cancers: 59 cases (27%) were positive, and the remaining 158 (73%) were negative [23]. They found a significant correlation between the lack of CD117 expression and lymph node metastasis (*p* < 0.0001), but not with tumor size or grade [23]. In keeping with our Kaplan-Meier analyses, they found a significant association in univariate analysis between the lack of CD117 expression and a worse DFS (*p* = 0.0041), but with multivariate analysis, the CD117 expression lost its significance (*p* = 0.31) [23]. CD117 has been found to be associated with the basal-like phenotype, but had no additive value in identifying these tumors by immunohistochemistry when EGFR or CK5 expression was present [5]; as this phenotype has great overlap with TNBCs, CD117 expression is likely to reflect the basal-like nature of the tumor, and this may also explain the lack of significance in multivariate analysis.

With CD166 staining, marginally significantly worse OS was identified for cases with focal staining, when compared to the negative cases (*p* = 0.05). Interestingly, the survival of cases with diffuse labeling with the antibody showed no statistical significance from negative cases, which can probably be explained by the low case number in the negative group. In TNBCs, the intensity of CD166 staining is lower than in other subtypes, and lower CD166 membranous labelling has been correlated with aggressive behavior [13]. Hein et al. examined 347 breast cancer patients’ TMA with IHC [24]. These cases contained no special type carcinomas, lobular carcinomas, “ductulolobular” carcinomas and others, and also included ER/PR-positive and -negative cases [24]. In 74 cases, the staining was negative, 136 cases had weak staining, 95 cases were moderately positive (these latter 2 categories were then combined into one as there was no significant difference between them), and finally 42 cases were strongly positive [24]. With Kaplan-Meier analysis, compared to the OS estimates of the weak/moderate category, they found an association between high ALCAM expression and a worse prognosis (*p* = 0.021) [24]. The differences in RFS were not significant (*p* = 0.279) [24]. The prognostic impact of this marker expression was higher in invasive ductal carcinomas (no special type) than in the other subtypes (OS *p* = 0.003; RFS *p* = 0.048) [24]. With univariate Cox regression analysis, in no special type carcinomas, ALCAM overexpression was associated with worse prognosis (OS: HR = 4.32, 95% CI: 1.61–11.56, *p* = 0.004, and the RFS: HR = 2.47, 95% CI: 1.17–5.22, *p* = 0.018), while in the multivariate analysis, with stage, nodal status, grade, ER status, the prognostic value of ALCAM expression was borderline, *p* = 0.050 [24].

In our study, ALDH1 expression was associated with shorter DFS, but not with shorter OS. Both cases with focal, (*p* = 0.013) and diffuse positivity (*p* = 0.043) had significantly worse DFS than cases without expression. The prognostic relevance of ALDH1 expression is supported by several literature data. Ginestier et al. examined the ALDH1 staining in TMAs of 481 breast cancers of different phenotypes from two different centers [25]. ALDH1 staining correlated with basal cytokeratin expression which is a common feature in TNBCs [25]. Like in our series, only a minority of the cases showed diffuse staining, the average labelling was 5%. The relative risk of death because of cancer in ALDH1-expressing and ALDH1-negative tumor cases was 1.76 (*p* < 0.028) [25]. ALDH1-positive cases had worse 5-year-OS (20% and 70% in the two centers, respectively) than ALDH1-negative ones (59% and 85%, respectively) [25]. Although our OS analysis did not show a prognostic effect, this was observed with the DFS analysis, the worse 5 year-DFS seen with ALDH1 staining was intermediate between the OS values of the two centers reported by Ginestier et al. [25]. Zhou et al examined different marker expressions, including ALDH1 expression by IHC in 31 TNBC and 89 non-TNBC cases [26]. The ALDH1 expression rate was significantly higher in TNBCs than in non-TNBCs (*p* = 0.015) and was associated with OS in both subsets; it turned out to be an independent prognostic factor reflecting poor prognosis [26]. Kida et al found 21% of 653 invasive BC core needle biopsy samples to be positive for ALDH1 by IHC, whereas the rate in TNBCs was somewhat higher (30%) [27]. Expression was correlated with larger tumor size (*p* < 0.001) (like in our study), clinical node metastasis (*p* = 0.004), higher clinical stage (*p* < 0.001), higher nuclear grade (*p* = 0.005), hormone receptor positivity (*p* < 0.001), and HER2 negativity (*p* < 0.001) [27]. ALDH1-expressing tumors had significantly shorter DFS (*p <* 0.001) and OS (*p* = 0.044), and ALDH1 proved to be an independent predictor for DFS (*p* = 0.033), but not OS [27]. Taking into consideration the subtypes of BCs, ALDH1 expression was associated with poor prognosis in luminal-type cancers, but not in TNBCs or HER2-positive tumors [27]. Ma F. et al examined ALDH1 expression by IHC in 158 TNBCs. Similarly to our results, somewhat more than half of the cases were positive, and expression was associated with tumor size (*p* = 0.02) and stage (*p* = 0.04). ALDH1-positive cases had shorter relapse-free survival (RFS) and OS [28]. The multivariate Cox regression showed that ALDH1 expression was an independent prognostic indicator for both RFS and OS in TNBCs [28].

With the evaluation of CD44 expression, we found a significant difference between the OS of negative and diffusely positive cases (*p* = 0.014). Interestingly, in the focally positive cases, the OS was 62% which is close to the OS of negative cases. Kim et al., in 2010, investigated the prognostic significance of CD24 and CD44 expression in breast cancer [29]. In their study, the 10-year-DFS was 62% for the cases lacking CD44 expression, and it was 73% for CD44-positive patients (*p* = 0.012) [29]. The 10 year-OS of CD44-negative cases was 68%, and that of CD44-positive cases was 78% (*p* = 0.013) [29]. In contrast, Collina F et al examined 160 TNBCs with IHC for several marker expressions, including CD44, and reported that the rare cytoplasmic staining was associated with metastasis and DFS [30]. Shadbad et al. made a systematic review to determine the prognostic value of CD44 expression in TNBCs [31]. Based on the 9 studies included, CD44 and CD44+/CD24-/low phenotype is associated with poor OS and DFS estimates [31]. The two above-quoted results [30, 31] are in contrast with ours, and this might be explained by the dual nature of the CD44 function detailed below [32].

Louderbough et al. evaluated 15 studies which were examining the correlation between CD44 expression and prognosis [32]. Based on their summary, in 4 studies, CD44 expression was associated with an unfavorable prognosis, in 6 studies the expression was related to a favorable prognosis, while in 2 studies it was neutral [32]. In 2 studies, the association between CD44 expression and prognosis was dependent on the variant expression [32]. The remaining 1 study did not evaluate the clinical outcome [32]. Based on their research, CD44 has a connection with both cancer progression inhibition and promotion [32]. The metastasis-suppressing activity of CD44 is increased by high molecular weight hyaluronan, while low molecular weight hyaluronan acts antagonistically [32]. Another important feature is that CD44 has differently spliced isoforms, and this can also explain how CD44^+^ tumor cells differently mediate biology; e.g., CD44v3 isoform is associated with increased cell migration and invasion [32]. The variants’ expression could also be a reason behind the differences in the favorable and unfavorable prognosis [32].

The present study has several limitations. Some marker expression subgroups were small (Supplementary Table S3), as discussed in connection with CD166 expression. The patients received different adjuvant therapies. Although most systemic treatments were acceptable according to guidelines at their time, including the denial of adjuvant treatment in some patients, this may have influenced survivals. Seven patients with neoadjuvant treatment without treatment effect on the tumor were also included, and this ineffective treatment might have also affected the outcome.

We attempted to compare the stem cell marker expression and the clinical outcome in TNBCs. With CD44-, CD117-and CD166-positive cancers, we found a significant effect of the expression of these markers on OS. In ALDH1- and CD117-positive cases, the staining differences showed a correlation with DFS. In all the other IHC markers, we could not identify significant correlations. CD117 was the only marker for which we found a correlation with both OS and DFS. According to the multivariate Cox-regression analysis, CD44, ALDH1 and the lymph node status showed a significant effect on prognosis. Owing to the controversial nature of the interpretation of the results, the role of CD44 expression in TNBC requires further investigation.

## Data Availability

The original contributions presented in the study are included in the article/Supplementary Material, further inquiries can be directed to the corresponding author.
